# Local Geometry, Structure and Electronic Resonances Enhancing the SFG Signal from CO on Ir Surfaces

**DOI:** 10.1021/acs.jpcc.5c02545

**Published:** 2025-07-01

**Authors:** Xia Li, Stefania Baronio, Susanne Gross, Thomas Haunold, Erik Vesselli, Günther Rupprechter

**Affiliations:** † Institute of Materials Chemistry, 27259 Technische Universität Wien, Getreidemarkt 9/BC, Vienna 1060, Austria; ‡ Physics Department, University of Trieste, Via Valerio 2, Trieste I-34127, Italy; § CNR—Istituto Officina dei Materiali (IOM), Area Science Park, SS 14 km 163.5, Basovizza I-34149, Trieste, Italy

## Abstract

Sum frequency generation (SFG) spectroscopy was used to study CO adsorption on smooth and rough Ir(111) single crystal surfaces, the cleanliness, composition, order and morphology of which were comprehensively characterized by Auger electron spectroscopy (AES), low energy ion scattering (LEIS), low energy electron diffraction (LEED), and scanning tunneling microscopy (STM). For CO adsorbed on Ir(111), the resonant SFG signal intensity associated with the internal C–O stretch mode was about eight times stronger on a rough termination than on a smooth surface. Herein, we thoroughly discuss the origin of this phenomenon and consider several possible contributing factors, including coverage and lateral interactions, molecular hyperpolarizability (IR dipole moment and Raman polarizability), adsorption geometry (tilt angle), Fermi resonances, adsorbate hot vibrational bands, and surface plasmons and electronic structure. It is concluded that the sputter-induced local roughness of the Ir surface (grains evidenced by STM) facilitates the light-induced excitation of localized surface plasmon resonances (LSPR), accounting for the observed signal enhancement.

## Graphical abstract

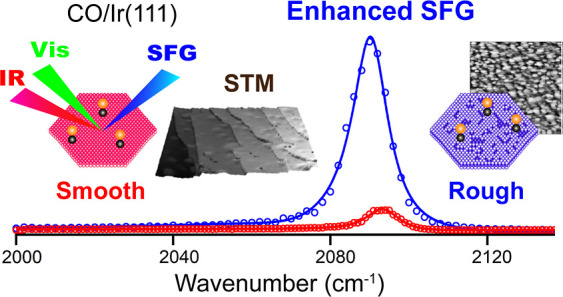

## 1 Introduction

The adsorption properties of CO on single-crystal metal surfaces are a core topic in model catalysis. Numerous studies in the past decades have investigated CO adsorption sites, geometries, desorption behavior and CO-induced surface reconstruction, primarily using ultrahigh vacuum (UHV)-based surface-sensitive spectroscopy and microscopy techniques.^1,2^ However, these methods typically suffer from a “pressure-gap”, as catalytic processes typically occur at pressures of 1 atm or above. Accordingly, second-order nonlinear sum frequency generation (SFG) spectroscopy, a photon-in and photon-out technique, can overcome this limitation when combined with a suitable UHV-to-high-pressure cell,^3,4^ due to the inherent surface sensitivity and selectivity of SFG, even at atmospheric gas pressure.

Recently, we carried out polarization-dependent (ppp and ssp) infrared-visible sum frequency generation (SFG) studies of CO adsorption on smooth^3^ and rough^4^ Ir(111) surfaces at various CO coverages. The surface coverage of CO was adjusted by varying either the CO pressure or the substrate temperature. Only on-top CO was detected, contributing with the fundamental band *v* = 0 → 1 transition to the resonant vibronic intensity,^5^ with the CO tilt angle highly dependent on coverage on the smooth surface, whereas it was only slightly coverage-dependent on the rough termination. On smooth Ir(111), CO molecules were oriented upright at low coverage, but tilted at high coverage,^3^ whereas on the rough surface, CO remained perpendicular to the surface.^4^

In addition, the spectral intensities of the on-top CO species differed significantly between smooth and rough Ir(111) surfaces: the smooth surface exhibited weaker ppp and stronger ssp intensities, while the rough surface showed an opposite behavior, with stronger ppp and weaker ssp intensities. This is in contrast to the case of a platinum (Pt) metal termination, for which the (ppp-) SFG signal has been reported to be larger on smooth surfaces than on rough ones.^6^ The signal enhancement in the ppp spectra was previously attributed to a combination of factors, including CO tilt angle, order, and coverage, but no detailed explanation has been provided so far.^4^ Herein, we carefully investigate the possible origin of the roughness-induced SFG intensity enhancement by considering several possible contributing factors, including the adsorbate bonding geometry, the local surface electronic structure, and vibrational contributions such as the excitation of CO hot bands and the vibrational coupling into Fermi resonances.

## 2 SFG Theory

Sum frequency generation (SFG) is an interface-sensitive second-order nonlinear optical technique. Under the electric-dipole approximation, SFG is forbidden in media with inversion symmetry, whereas allowed at surfaces or interfaces where the inversion symmetry is broken.^7–11^ To generate a vibronic IR-Vis SFG signal, a visible input beam (ω_Vis_) and an infrared input beam (ω_IR_) are spatially and temporally overlapped at the sample surface, either in a copropagating or in a counter-propagating geometry. An interface-specific output SFG signal at the sum frequency (ω_SFG_ = ω_Vis_ + ω_IR_) is then generated in the phase-matching direction. The impinging beams can be selectively p- or s-polarized, and the generated SFG radiation can be decomposed into p- and s-components as well, with reference to the polarization direction of the optical field parallel and perpendicular to the incidence plane, respectively, in order to sample different components of the nonlinear susceptibility tensor. Thus, SFG can be measured in different polarization combinations (p or s), with the “label” arranged in the order of increasing wavelength, e.g., ssp (s-SFG, s-visible, and p-IR), ppp, sps, and pss. For metal surfaces, the IR laser beam must always be p-polarized, as the surface electric-field of an s-polarized IR laser beam is screened by the conduction electrons of the metal, while, due to the lower dielectric constants of metals in the visible region, the SFG and visible beams have a surface electric field that is less effectively screened.^12^ Therefore, for molecules adsorbing on metal surfaces, only ssp and ppp polarization combinations yield detectable signals.^1,2,13,14^

The ppp and ssp spectral intensities can be expressed in eqs 1 and 2 for molecules with *C*_∞ν_ symmetry (e.g., CO), respectively. As one can see, the SFG intensity is proportional to the square of χ_eff_^(2)^, which is the effective second-order nonlinear susceptibility of surface molecules.

1 $$I_{ppp}\propto {\left|\chi_{eff,ppp}^{\left(2\right)}\right|}^{2}={\left|{f_{xxz}\chi }_{xxz}^{\left(2\right)}+{f_{xzx}\chi }_{xzx}^{\left(2\right)}+{f_{zxx}\chi }_{zxx}^{\left(2\right)}+{f_{zzz}\chi }_{zzz}^{\left(2\right)}\right|}^{2}={\left|f_{xxz}\right|}^{2}{\left|\frac{1}{2}N_{s}\beta_{ccc}^{\left(2\right)}\left[\left(1+R\right)\left\langle \cos\theta \right\rangle -\left(1-R\right)\left\langle \cos^{3}\theta \right\rangle \right]\right|}^{2}+{\left|f_{xzx}\right|}^{2}{\left|\frac{1}{2}N_{s}\beta_{ccc}^{\left(2\right)}\left[\left(1-R\right)\left\langle \cos\theta \right\rangle -\left(1-R\right)\left\langle \cos^{3}\theta \right\rangle \right]\right|}^{2}+{\left|f_{zxx}\right|}^{2}{\left|\frac{1}{2}N_{s}\beta_{ccc}^{\left(2\right)}\left[\left(1-R\right)\left\langle \cos\theta \right\rangle -\left(1-R\right)\left\langle \cos^{3}\theta \right\rangle \right]\right|}^{2}+{\left|f_{zzz}\right|}^{2}{\left|N_{s}\beta_{ccc}^{\left(2\right)}\left[R\left\langle \cos\theta \right\rangle +\left(1-R\right)\left\langle \cos^{3}\theta \right\rangle \right]\right|}^{2}$$

2 $$I_{ssp}\propto {\left|\chi_{eff,ssp}^{\left(2\right)}\right|}^{2}={\left|{f_{yyz}\chi }_{yyz}^{\left(2\right)}\right|}^{2}={\left|f_{yyz}\right|}^{2}{\left|\frac{1}{2}N_{s}\beta_{ccc}^{\left(2\right)}\left[\left(1+R\right)\left\langle \cos\theta \right\rangle -\left(1-R\right)\left\langle \cos^{3}\theta \right\rangle \right]\right|}^{2}$$

*f*_*ijk*_ is a constant for molecules under selected experimental conditions, as it is only a function of incident angles of the fields, their polarizations, and the frequency-dependent refractive indices of bulk media and the interface layer.^15^ χ_*ijk*_^(2)^ is the second-order macroscopic susceptibility, which is a function of the effective surface density *N*_s_ of molecules, the molecular tilt angle θ (e.g., the angle between the CO molecular axis and the surface normal), molecular microscopic hyperpolarizability β_ccc_^(2)^ and molecular hyperpolarizability ratio *R* = β_aac_^(2)^/β_ccc_^(2)^ = β_bbc_^(2)^/β_ccc_^(2)^. $\beta_{i^{\prime }j^{\prime }k^{\prime }}^{\left(2\right)}=-\alpha_{i^{\prime }j^{\prime }}^{\prime }\mu_{k^{\prime }}^{\prime }/2\varepsilon_{0}\omega_{q}$ with $\alpha_{i^{\prime }j^{\prime }}^{\prime }$ , $\mu_{k^{\prime }}^{\prime }$ , ω_q_ and ε_0_ being the derivatives of the molecular Raman polarizability and IR transition dipole moment with respect to the qth vibrational mode, resonant frequency of the qth mode, and the static optical dielectric constant, respectively. Thus, for a vibrational mode to be SFG active, it must simultaneously satisfy both Raman and IR selection rules.^7–10^ By using the ratio *I*_ppp_/*I*_ssp_, which depends only on *R* and the tilt angle θ, one can determine θ if *R* is known, and vice versa.^3,4,16,17^ For more information regarding the fundamental SFG theory and the orientation analysis, one can refer to our recent review paper.^2^

## 3 Experimental Section

### 3.1 Chemical

CO of purity 4.7 (99.997%) from Messer Austria and 3.0 from SIAD were used.

### 3.2 Pretreatments and Surface Characterization of Ir(111)

#### 3.2.1 Pretreatments

Two different Ir(111) single crystals (8 mm φ, 2 and 1.5 mm thickness) were used for the SFG measurements in Vienna and Trieste, pretreated in ultrahigh vacuum (UHV) using standard cycles of Ar^+^-ion sputtering (1.2 keV beam energy, 10^–6^ mbar Ar, 30 min, 300 K), oxidation (1 × 10^–7^ mbar O_2_, 30 min, 800 K), and annealing (UHV, 30 min, 1050 K). The freshly pretreated “smooth” Ir(111) was directly transferred from the UHV chamber to the SFG spectroscopy cell under UHV, avoiding contaminations. A “rough” Ir(111) surface was obtained by sputtering the “smooth” surface with normal angle of incidence using a beam energy of 1.2 keV at 10^–6^ mbar Ar for 40 min at 300 K without subsequent annealing, similarly to the recipe yielding a high step density on Pd(111).^18^ Upon increasing the sputtering parameters, including beam energy (voltage), argon (Ar) gas pressure, and sputtering time, they all lead to higher surface roughness.

#### 3.2.2 Surface Characterization

Surface cleanliness was confirmed by Auger electron spectroscopy (AES) and low energy ion scattering (LEIS). Surface order and morphology were determined by low energy electron diffraction (LEED) and scanning tunneling microscopy (STM), respectively. For AES spectra and LEED patterns of Ir(111) surfaces refer to refs 4 and 5 published by the Vienna and Trieste groups. Unlike Ir(100)^19–21^ and Ir(110)^22^ surfaces, CO pressure- and substrate temperature-dependent LEED patterns on Ir(111) surfaces showed no evidence of surface roughening or reconstruction,^4^ in line with Ir(111) being considered the most stable surface.^23^ The setup for LEIS and STM consisted of two connected separate UHV stainless steel chambers (Vienna), at a base pressure of ≤4 × 10^–10^ mbar and 9 × 10^–10^ mbar, respectively.^24^ The spectroscopy chamber was equipped with a Phoibos 100 hemispherical energy analyzer (EA) featuring a multichannel plate detector for detection as well as a SPECS IQE 12/38 ion source for LEIS, operating with He^+^ ions at a kinetic energy of 1 keV, a helium backpressure of 2 × 10^–7^ mbar, and a scattering angle of 135°. STM experiments were carried out at room temperature using an Aarhus-type STM 150 SPECS setup.^25^

### 3.3 SFG Spectroscopy

The SFG measurements were performed in two different, but equivalent setups at TU Wien (Vienna)^3,6,26^ and at the University of Trieste^5^ to confirm reproducibility and exclude artifacts. For the same reason, we used different Ir(111) single crystals. In the former case (Vienna setup), SFG spectra were measured in a “high pressure cell” at pressures ranging from 2.5 × 10^–8^ to 1 mbar and temperatures from 100 to 800 K.^3,6,26^ The SFG measurements were conducted using a 20 ps mode-locked Nd:YAG laser system (PL2241, EKSPLA, Lithuania) with fundamental radiation at 1064 nm (30 mJ/pulse, 50 Hz repetition rate). This laser system generally generates a visible beam at 532 nm and a tunable infrared (IR) beam in the range of 2.3–10 μm. The visible and IR beams were directed in a copropagating geometry toward the Ir(111) surface, with incidence angles of 58.5° and 55°, respectively. The pulse energy was 30 ± 5 μJ for the visible beam and 90–130 μJ for the IR Beam. The SFG signal was detected in the reflection direction using a photomultiplier tube (PMT). SFG spectra were measured in both ppp and ssp polarization combinations and normalized by the intensities of the impinging visible and IR beams (detected simultaneously in each spectral run). In the second setup (Trieste) a similar EKSPLA setup (PL2231) was employed,^5^ coupled with a high-pressure measurement cell that is directly connected to a UHV sample preparation and characterization system, hosting standard surface science techniques and instrumentation (ion gun, gas lines, QMS (quadrupole mass spectrometer), LEED, AES, etc.). Conditions for sample preparation in UHV and for the measurements in the high-pressure cell were similar for the two setups.

## 4 Results and Discussion

### 4.1 LEIS and STM Characterization of Smooth and Rough Ir(111)

After multiple cleaning cycles of the Ir(111) single crystal and surface characterization by LEIS (Figure 1), STM measurements were performed both for annealed and sputtered surfaces. Figure 2a,b illustrates the smooth Ir(111) surface consisting of relatively large terraces. Using the Gwyddion software (version 2.68),^27^ the lateral correlation length, calculated perpendicular and parallel to the terrace, is 14.2 and 10.6 nm, respectively. The root-mean-square (RMS) roughness is 56 pm along the terraces and 122 pm across them, as expected for stepped single crystal surfaces. The lighter spots in Figure 2a,b likely originate from subsurface contaminants such as carbon, oxygen or sulfur (as reported for Pd(111)^28^), or subsurface argon nanobubbles.^29^ Note that this assignment is supported by the absence of surface carbon in LEIS (Figure 1), with solely iridium (Ir, ∼900 eV) and oxygen (O, ∼400 eV) observed, as reported previously for a clean surface.^24^ The LEIS peak positions were determined according to their high-energy “foot”, via the LEIS energy calculator.^30^ Intriguingly, for the sputtered surface, STM images clearly revealed many small three-dimensional rough mounds (Ir grains/nanoparticles) ranging from 3 to 8 nm in size (Figure 2c–e), with short correlation lengths (2.9 nm perpendicular and 2.3 nm parallel) and a high RMS roughness of approximately 124 pm in both directions, indicating a disordered nanostructured surface. LEIS is not meaningful after sputtering due to the strong surface roughness. Corresponding LEED, AES and XPS characterization of smooth and rough Ir were already reported in ref 4.

**Figure 1:**
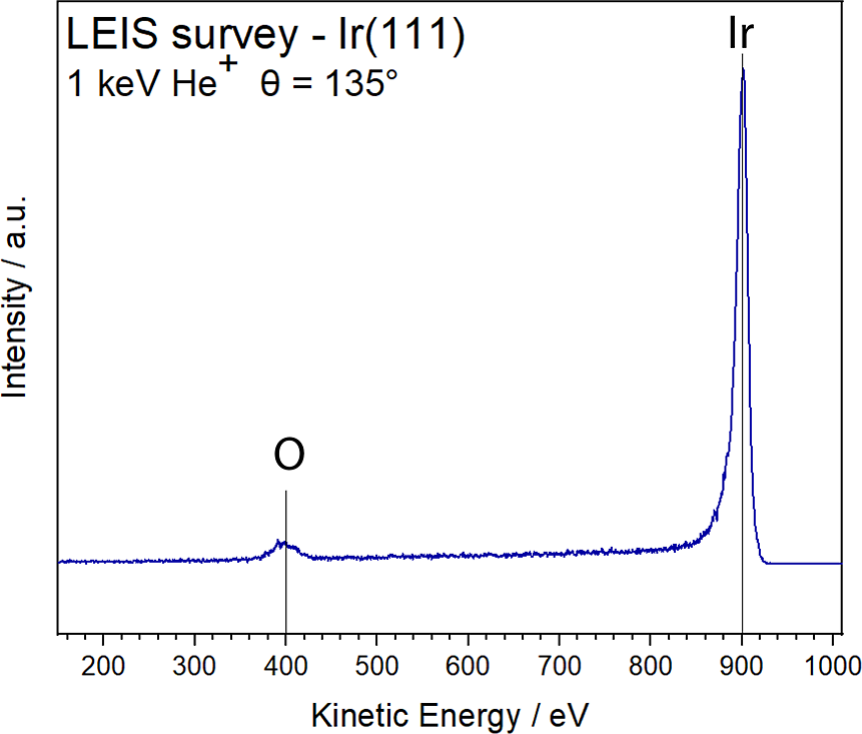
LEIS of the clean and smooth Ir(111) surface, indicating the presence of only iridium (∼900 eV) and oxygen (at ∼400 eV) atoms without any traces of carbon.

**Figure 2:**
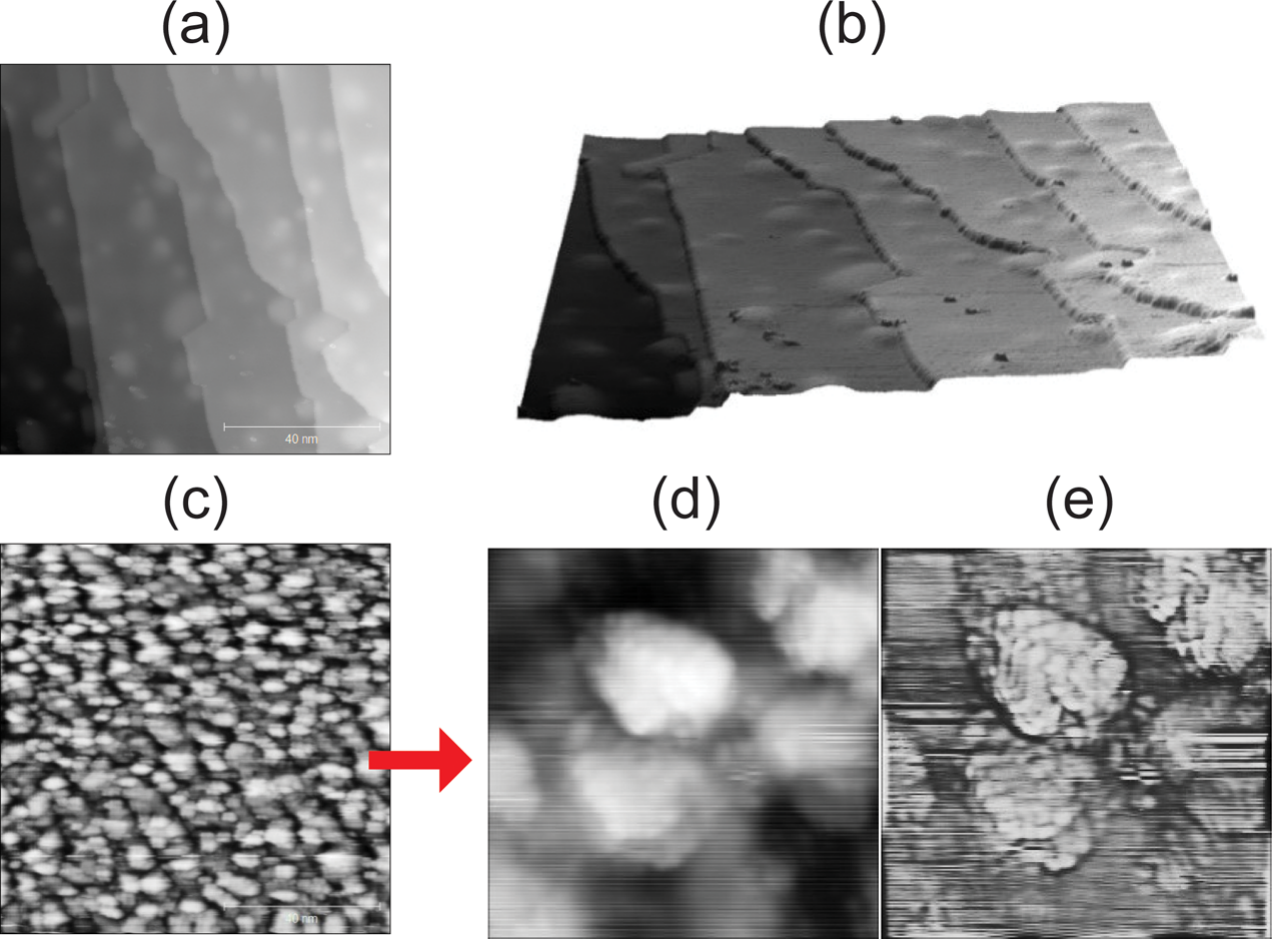
STM topography of (a) the smooth Ir(111) surface following Ar^+^ sputtering, oxidation, and subsequent annealing in UHV (100 × 100 nm^2^), accompanied by (b) a 3D view highlighting the terraces and steps. (c) The rough surface after Ar^+^ sputtering without subsequent annealing (100 × 100 nm^2^), showing the formation of small three-dimensional islands, likely featuring a high density of steps, as previously reported for sputtered Pd(111) surfaces.^18^ (d) Magnified view (10 × 10 nm^2^) of the mounds in (c) . Due to the high noise level, the steps are not clearly resolved, but better visible after applying a high-pass filter, as shown in (e). Tunneling parameters: (a,b) *I*_t_ = 0.66 nA, *V*_t_ = 700.1 mV, (c) *I*_t_ = 1.3 nA, *V*_t_ = 700.1 mV, (d,e) *I*_t_ = 0.78 nA, *V*_t_ = 699.8 mV.

### 4.2 SFG Spectra of CO Adsorption on Smooth and Rough Ir(111)

Figure 3 shows the ppp and ssp spectra of CO (1.0 mbar at 300 K) adsorbed on smooth and rough surfaces at room temperature (acquired in the TU Wien lab). Note that the ssp spectra intensity is several hundred times weaker, resulting in a poorer signal-to-noise ratio. All peaks refer to a CO stretch mode associated with the fundamental transition (*v* = 0 →1). On the smooth surface, the peak at 2094 cm^–1^ is attributed to on-top CO (corresponding to a saturation coverage of 0.77 ML), but on rough surfaces it slightly shifts to lower wavenumbers (2090 cm^–1^, corresponding to 0.70 ML coverage), indicating a close to negligible increase in the CO binding strength to the substrate.^4,6^ The actual surface CO coverage was deduced based on the relationship between CO coverage and the IR peak position obtained in a combined IRAS/TPD study of CO on Ir(111).^31^

**Figure 3:**
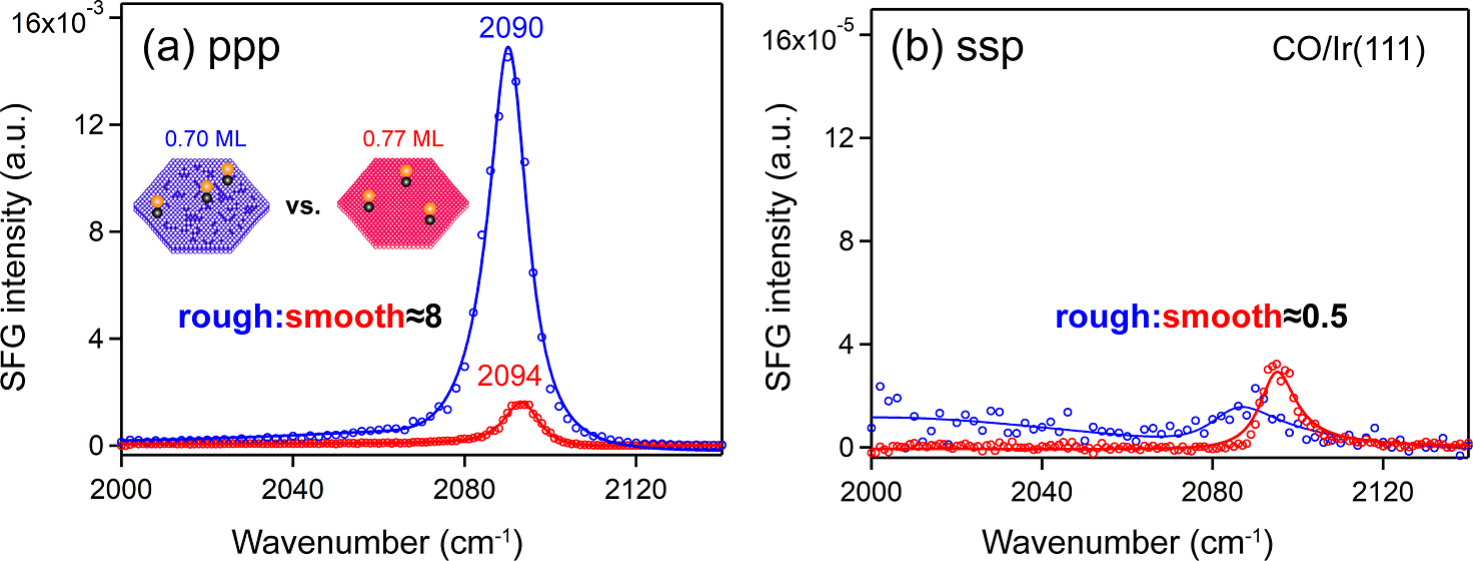
SFG spectra of on-top CO on Ir(111) single crystal. 1.0 mbar CO adsorbed on a smooth (red) and rough (blue) Ir(111) surface at 300 K with (a) ppp and (b) ssp polarization combinations. Spectra were acquired at TU Wien.

The most surprising and striking feature in Figure 3 is revealed by comparing the spectral intensities from smooth and sputtered rough Ir(111), the ppp intensity on the rough surface being eight times larger (Figure 3a), while the corresponding ssp intensity is about two times weaker (Figure 3b). This intensity enhancement in ppp spectra was also reported in ref 4, both for various CO coverages and substrate temperatures. Clearly, the *I*_ppp_/*I*_ssp_ intensity ratio differs strongly (rough vs smooth: 820 vs 36), suggesting at least a contribution from a different CO tilt angle,^3,4^ while not excluding an electronic effect as well.

To verify the strong intensity enhancement, additional spectra were collected for CO adsorption on both smooth and rough Ir(111) surfaces under various CO pressures at 300 K at different time (Figure S1, TU Wien), and also using a different Ir(111) single crystal and SFG spectrometer (University of Trieste), as shown in Figure 4. Alike Figure 3, an intensity enhancement was observed on the rough surface as the CO pressure increased from 10^–7^ to the mbar range. For instance, in Figure 4, at 0.1 mbar CO (blue), the ppp-intensity on the rough surface was more than 8 times stronger than on the smooth surface. This effect was observed also after the CO gas phase had been pumped out (green), indicating irreversible CO adsorption at room temperature and ruling out a contribution from the interaction of the layer with the gas phase and from metastable adsorption configurations. Compared to the rough surfaces, the “tail” at high wavenumbers on the smooth surfaces is merely background with no associated features.

**Figure 4:**
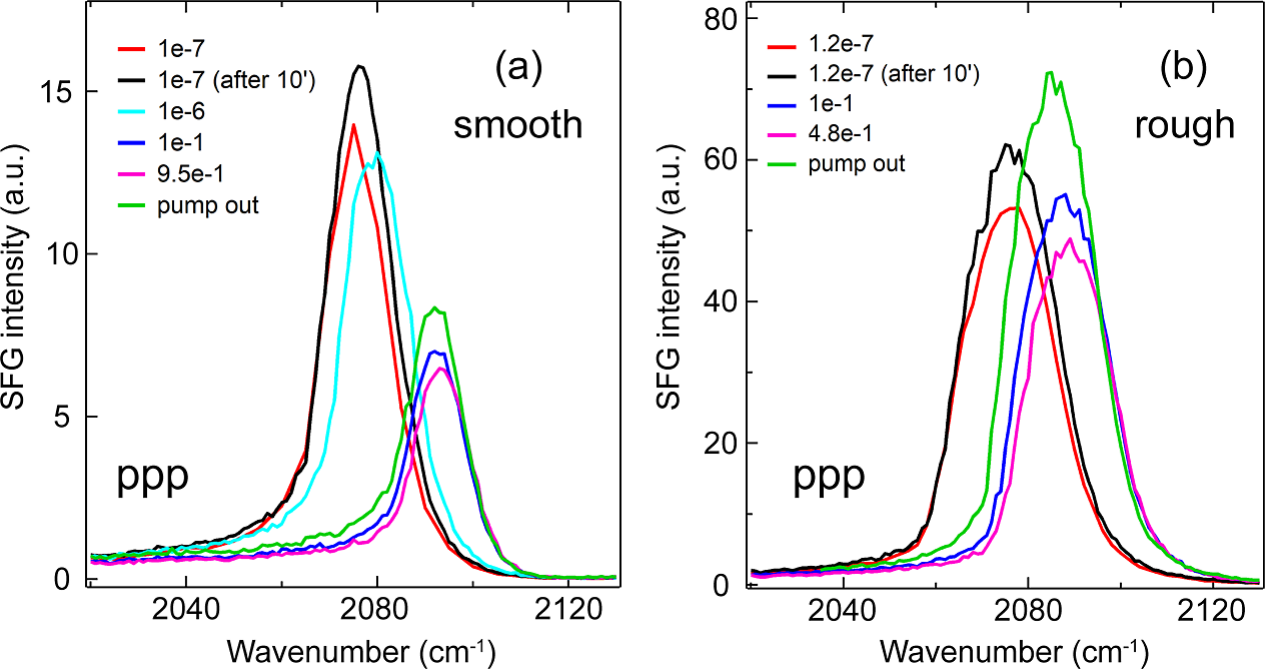
ppp-SFG spectra of on-top CO on Ir(111) single crystal at 300 K: (a) on smooth surface and (b) on rough surface. Spectra acquired at University of Trieste.

On rough surfaces, the full width at half-maximum (fwhm) of the on-top CO peak was approximately 2 cm^–1^ larger (∼10 vs 8 cm^–1^ on smooth surface). In general, two mechanisms are typically held responsible for line broadening:^32,33^ one is the energy exchange between fundamental and excited oscillators (Lorentzian width associated with the dephasing time), and the other is inhomogeneous (Gaussian) broadening due to surface disorder. As shown in Figure 3, a 2 cm^–1^ increase on the rough surface suggests only a slight increase of the energy exchange rate or a minimal contribution from the layer disorder. Anyway, the two contributions (Lorentzian and Gaussian) cannot be deconvoluted beyond the statistical uncertainty limit due to their small values.

In the following, we will address a number of potential factors influencing the spectral intensity enhancement: CO coverage, molecular hyperpolarizability (β_ccc_^(2)^ and its ratio *R* = β_aac_^(2)^/β_ccc_^(2)^ = β_bbc_^(2)^/β_ccc_^(2)^), CO tilt angle, which are based on eqs 1 and 2, and other vibrational contributions such as the excitation of CO hot bands, Fermi resonances, as well as surface plasmon resonance (SPR) effects.

### 4.3 Effects of CO Coverage, Hyperpolarizability, and Tilt Angle on the SFG Intensity

#### 4.3.1 Coverage

As shown in eqs 1 and 2, the SFG intensity is proportional to the square of the effective surface density *N*_s_ of CO molecules. Thus, because the CO coverage is lower on the rough surface (0.7 ML vs 0.77 ML, Figure 3) at room temperature,^4^ both its ppp and ssp intensities should only be 80% (0.70^2^/0.77^2^) of the smooth surface case (Table 1). For ppp, this is opposite to the observed behavior, indicating that there must be another origin, thus invalidating the coverage-dependent origin.

**Table 1:** Effect of N_s_ and Tilt Angle of Surface CO Molecules on ppp and ssp Intensities

	I_ppp,rough_/I_ppp,smooth_	I_ssp,rough_/I_ssp,smooth_
**experimental**	**8.0**	**0.5**
effect of tilt angle (θ)	2.5	0.1
effect of square of coverage (i.e., N_s_^2^)	0.8	0.8
**total effect of θ and N**_**s**_^**2**^	**2.0**	**0.08**

The coverage-dependent CO adsorption on both smooth and rough Ir(111) surfaces has been systematically studied in refs 3 and 4 . It was found that on the smooth surface, a significant decrease in ppp-SFG signal intensity was observed with increasing coverage/pressure (refs 3 and 4 and Figure 4). This was attributed to large tilt angles of CO molecules at high coverages.^3^ In contrast, on the rough surface, the spectral intensity decreased comparably less (ref 4 and Figure 4), as the CO tilt angle showed only a slight dependence on coverage.^4^

#### 4.3.2 CO Hyperpolarizability Ratio *R*

As discussed in detail in refs 3 and 4 , the *R*-values for CO on the rough and smooth Ir(111) surfaces were determined to be 0.06 and 0.08, respectively, using the highest ratios of *I*_ppp_/*I*_ssp_ (820 for the rough surface and 520 for the smooth surface), based on the simulated *I*_ppp_/*I*_ssp_ curves versus CO tilt angle using different *R*-values. Figure 5 shows the calculated Ippp and Issp as a function of the CO tilt angle, with *R* = 0.06 (blue) for the rough and *R* = 0.08 (red) for the smooth surfaces, respectively. It is evident that there is only a slight variation in both ppp and ssp intensities, with the rough surface showing slightly lower values. This also rules out the *R*-value being the cause of the enhancement and discards the second hypothesis as well. Moreover, based on eqs 1 and 2, the molecular hyperpolarizability (β_ccc_^(2)^) simultaneously affects both ppp and ssp spectral intensities, which implies an increase on the rough surfaces, but this needs further theoretical calculation.

**Figure 5:**
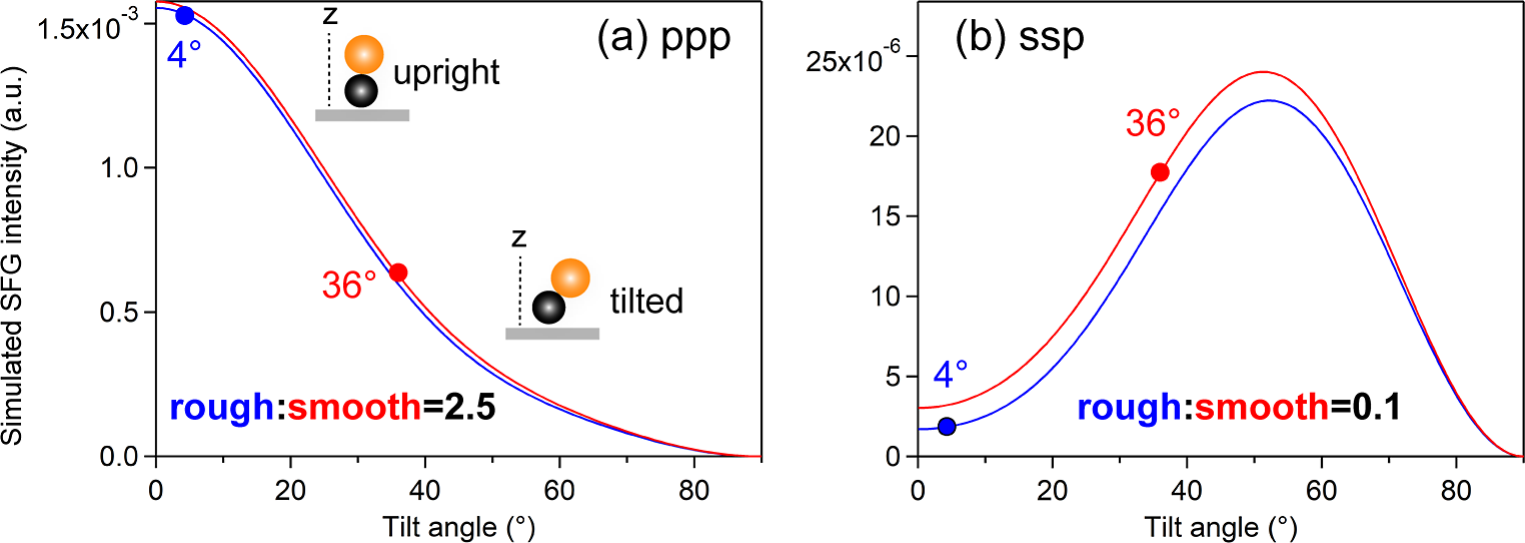
Simulated SFG intensity as a function of CO tilt angle: (a) ppp and (b) ssp. The CO molecular hyperpolarizability ratio *R* was 0.06 and 0.08 for rough (blue) and smooth (red) Ir surfaces, respectively.^4^ *N*_s_ and β_ccc_^(2)^ were assumed to be 1. The values of ${\left|f_{xxz}\right|}^{2}$ = 3.3 × 10^–3^, ${\left|f_{xzx}\right|}^{2}$ = 2.0 × 10^–3^, ${\left|f_{zxx}\right|}^{2}$ = 2.6 × 10^–3^, ${\left|f_{zzz}\right|}^{2}$ = 1.5 × 10^–3^, and ${\left|f_{yyz}\right|}^{2}$ = 4.6 × 10^–3^ were calculated using the same parameters, including known incidence/refraction angles, refractive indices, wavelengths of incident visible and IR and output SFG, as described in ref 3.

#### 4.3.3 CO Tilt Angle (θ)

As shown in Figure 5, the calculated ppp intensity decreases monotonously with increasing tilt angle θ (Figure 5a), while the ssp intensity gradually increases with increasing θ up to approximately 50°, after which it decreases (Figure 5b). In our recent work,^4^ the CO tilt angles at 0.70 ML on the rough surface and at 0.77 ML on the smooth surface were reported to be 4° (upright) and 36° (tilted) with respect to the surface normal, respectively. The ppp intensity (Figure 5a) at θ = 4° (blue line) is 2.5 times larger than that at θ = 36° (red line), whereas the ssp intensity (Figure 5b) at θ = 4° is 0.1 times smaller than that at θ = 36°. Taking into account both the contributions of tilt angle and coverage, the ppp intensity becomes twice stronger on the rough surface (i.e., I_ppp,rough_/I_ppp,smooth_ = 2.0), which is much less than the experimentally observed value of I_ppp,rough_/I_ppp,smooth_ = 8.0. Similarly, the calculated value of I_ssp,rough_/I_ssp,smooth_ is 0.08, which is again much smaller than the experimental value of 0.5 (Table 1). Also in this case, i.e. the contributions from the CO adsorption tilt angle, reasonable values do not account for the observed deviation.

### 4.4 Vibronic Contributions

#### 4.4.1 Fermi Resonance

Fermi resonances can affect the CO fundamental stretching mode. Fermi resonance refers to the coupling between a fundamental vibrational mode (*v* = 0 → 1) and either an overtone or a combination band, resulting in a shift in vibrational frequency and a change in spectral intensity. Overtone transitions occur when a mode (e.g., stretching, bending) is stepwise excited from the ground state (*v* = 0) beyond the fundamental vibrational state (*v* ≥ 2). Combination bands are observed when two or more fundamental vibrations (e.g., stretching + deformation/bending,^34^ bending + libration^35^) are excited simultaneously. A combination band can either be a sum of two frequency bands or a difference band.

When two vibrational modes have similar energies and interact strongly, Fermi resonance occurs and leads to a splitting or shift in peaks. For example, Fermi-resonance occurs when the OH fundamental stretching band overlaps with the first overtone of the bending mode in the H-bonded stretching region (3000–3800 cm^–1^). The Fermi resonance was crucial in the theoretical simulation of IR and Raman spectra of liquid water.^36^ Similarly, a Fermi resonance doublet of bridge-bonded ^12^C^16^O was observed via low-frequency IR spectra due to Fermi resonance coupling of the Pt–CO stretching mode (385 cm^–1^) and a difference combination band (frustrated translation at 510 cm^–1^ minus frustrated rotation at 133 cm^–1^), resulting in a second peak at 378 cm^–1^ with a 1:1 intensity ratio.^37^ However, based on 7 cm^–1^ isotopic frequency shifts in both cases (^12^C^16^O ↔^13^C^16^O; ^12^C^18^O ↔^13^C^18^O), no Fermi resonance-induced frequency shift was deduced, as commented by Jakob.^38^ Later, far-IR synchrotron radiation spectroscopy of CO adsorption on Pt(111) also showed no evidence of Fermi resonance-induced splitting of the absorption band assigned to the bridge bonded species.^39^

In any case, two experimental observations are characteristic of Fermi resonance mixing of modes: (i) an intensity gain of the (usually) weak combination band and an intensity loss of the strong fundamental mode, and (ii) a frequency shift of both component modes.^38^ Therefore, the enhancement observed in the fundamental stretching mode of CO (Figure 3) can clearly not result from a Fermi resonance.

#### 4.4.2 Hot Bands

Hot bands or hot transitions (e. g., *v* = *n* → *n* + 1, with *n* ≥ 1) are observed when the anharmonic vibrational transition ladder is climbed by multiple rungs through multiple, serial excitations. The anharmonic contribution to the potential energy profile of the vibrational mode yields progressive red shifts to the observed frequencies of the hot bands with respect to the fundamental (*v* = 0 → 1) transition, so that hot bands would be distinguishable.^40,41^ In broadband SFG, the high-intensity ultrashort infrared pulse (femtosecond-IR) allows access to hot band transitions.^42^ The simultaneous observation of both the fundamental and the subsequent hot transitions of CO on surfaces such as Ru(001),^42–44^ oxygen-covered Ru(001)^43^ and Ir(111)^33^ has thus been achieved by broadband SFG. For example, at a low CO coverage (0.07 ML) and low temperature (100 K) on Ir(111), with sufficient femtosecond IR pulse energy, a hot-band peak of on-top CO at a frequency of 2014.2 cm^–1^ becomes visible, with a 26.8 cm^–1^ anharmonic frequency red shift from the fundamental transition at 2041.0 cm^–1^.^33^ With increasing CO coverage, both the fundamental and hot bands showed a significant blue-shift in frequency and increase in line width, but with stronger effects on the hot band spectroscopic line. The change in CO frequency with coverage was explained by dipole–dipole coupling, with a stronger blue shift for the hot band as a result of a higher dipole moment of the *v* = 1 → 2 transition, while the change in line width with coverage was mainly caused by inhomogeneity of the CO layer accompanied by dipole-coupling induced line narrowing.^33^ Already at a slightly higher coverage (0.11 ML), the hot band was no longer discernible as a separate peak, though its contribution to the SFG spectra could be followed up to 0.25 ML. Due to intermolecular coupling, the hot band merged into the fundamental line at high coverage also for CO on Pt(111).^45^ Similarly, the hot-band peak of on-top CO on Ru(001) was only reported at very low coverage (below 0.02 ML, 95 K).^42–44^ After the unresolved hot and fundamental bands merged into a single peak, the CO peak intensity on Ir(111) increased.^33^ In Figures 3 and 4, all spectra on both rough and smooth surfaces were measured at coverages higher than 0.4 ML, where strong dipole coupling occurs. Since the observation of hot bands is limited to very low coverages, where intermolecular coupling is negligible,^43^ it is unlikely that the contribution from the hot band to the intensity enhancement is of any significance in the present case, particularly in the ppp spectra.

Recently, hot and fundamental bands were resolved by scanning mode SFG for CO adsorption on single metal atoms embedded in a 2D metal–organic framework.^46,47^ The single cobalt (Co) atoms were ∼1.5 to 2 nm apart, ruling out any contribution from direct lateral interactions and involving, instead, electron–phonon coupling, relaxation, and magnetic mechanisms. In that case, by varying the Co atom coverage (in the 1% ML range), the progressive evolution of the metal atom coordination in the network led to a corresponding tuning of the local electronic configuration and of the Co oxidation state. Interestingly, the amplitude of the internal C–O stretch resonant SFG signal of CO/Co was found to vary within 5-fold and 2-fold ranges for the fundamental and the hot bands, respectively. Thus, in this latter case, while an intensity enhancement was clearly observed when exciting both fundamental and hot bands, its origin was not related with the vibrational ladder transitions, but with a modification of the local electronic structure of the binding sites. These observations point to the direction of an electronic origin of the observed intensity increase for the rough Ir(111) termination, associated with plasmonic resonances of the metal termination and with a locally modified electronic structure due to the surface defects induced by the ion bombardment. Thus, these latter causes need further attention.

#### 4.4.3 Surface Plasmon Resonance (SPR)

The role of the surface electronic structure in both SFG phase and amplitude modulation has already been thoroughly addressed in previous work by Busson for the case of gold.^48–50^ It was found that surface and bulk contributions by free and bound electrons, both overlapping or spilling out of the bulk, significantly affect the resonant SFG process. To observe these effects, different complementary approaches can be adopted: tuning of the visible beam energy, as for the mentioned investigation on gold,^48–50^ or tuning of the local electronic structure, as for the mentioned single Co atoms^46,47^ and for our present case (roughening) as well.

As described in Section 3.2, the rough Ir surface was generated by Ar^+^-ion sputtering^18^ of a freshly prepared, well-ordered (smooth) (111) termination. When energetic ions impinge on a surface, they induce both geometric (cf. Figure 2c–e) and electronic structural changes, such as the creation of defects, surface roughening, local corrugation associated with ion implantation or the generation of nanostructures (throughs, pyramids, islands) and even of metal nanoparticles (NPs),^51,52^ alterations in surface stoichiometry due to different sputtering cross sections between different elements when the target is an alloy, a decrease in the optical band gap,^53^ and modifications of the electric conductance.^54^ Specifically, ion-sputtering-induced self-organized nanostructures can manifest as dots, holes, islands (e.g., dots + holes) or ripples, depending on the ion energy and incidence angle with respect to both the surface normal and the surface crystallographic directions.^51,52^

In the case of metallic nanoparticles with a size smaller than the wavelength of the impinging radiation, a significant enhancement of collective oscillations of surface electrons can be induced if they are illuminated with radiation with a frequency which is resonant with the plasmonic frequency. This effect is addressed as localized surface plasmon resonance (LSPR), which depends on the size of the surface nanoparticles/islands. The LSPR is responsible for an electromagnetic-field enhancement that leads to surface-enhanced spectroscopic processes.^55–58^ More in general, the same effect can occur in the presence of nanostructured surfaces, cavities and interfaces,^59^ with quantum mechanical effects such as nonlocal screening and electron tunneling affecting the frequency and lifetime of the LSPR as well as the local field enhancement.^60^ On Cu(111), it was observed that, starting from a single Cu adatom and proceeding to larger islands, the formation of a variety of quantum states takes place, progressively merging into the well-known two-dimensional Shockley surface state in the limit of extended islands.^61^ On Ni(111), standing-waves patterns due to the confinement of a Shockley-like surface state were observed on artificial nanoscale triangular islands assembled to the purpose.^62^

In general, the electronic properties of these systems depend on (and can be in principle be controlled by) the balance between the confinement and the perturbation of the surface states caused by the hosted nanostructures.^63^ All of this supports the idea that a nanopatterned, defective, or more generally speaking, rough surface with nanometer-size structures hosts these electronic states. A large variety of vibrational spectroscopies relying on surface plasmon-enhanced (SE) effects has been reported, including linear spectroscopies such as surface-enhanced IR (SEIR),^55,56,64–68^ surface-enhanced Raman scattering (SERS),^57,58,69–71^ surface-enhanced anti-Stokes Raman scattering,^58^ and surface-enhanced hyper-Raman scattering,^72^ as well as nonlinear spectroscopies—such as surface-enhanced femtosecond stimulated Raman scattering (FSRS),^73^ and surface-enhanced SFG (SESFG).^58,74–81^ For example, SESFG has been (mainly) observed in studies of silver and gold nanoparticles, such as copper phthalocyanine films on silver,^74^ 1-dodecanethiol (DDT) films on Au nanoparticles^76^ or nanopillars,^78^ thiophenol adsorption^77^ on Au nanoparticles, and electrochemical reactions at Au electrodes in aqueous solution and at a thiol self-assembled monolayer covered gold electrode.^79^

These surface-enhanced techniques have proven that the vibrational signals of molecules can be enhanced by several orders of magnitude when the molecules are located at or close to the surface of noble metal nanostructures with proper electronic configurations. In principle, both SERS and SEIR effects can contribute to the observed increase of SFG intensity, since the SFG signal amplification depends on the enhancement of the Raman activity in the visible range (SERS), and on that of the IR activity.^58^ Tian and Ren^82^ gave a detailed account of surface roughening procedures for electrodes of different metals that result in good-quality SERS-active electrode surfaces made from Pt, Ni, Co, Fe, Pd, Rh, and Ru. As an example, we recall that the intensity of vibrational bands in an SESFG spectrum has been reported to be enhanced by one to five orders magnitude, both in ppp and ssp polarization combinations, when the visible incident beam energy crosses the typical LSPR energy of metal nanoparticles.^74–81^ The carbonyl peak for air/PMMA (poly­(methyl methacrylate)/Au was more than 4 times stronger (4.2 times in ppp and 4.8 times in ssp) than that of the air/PMMA interface owing to the LSPR induced by gold nanoparticles.^81^ The ssp-SFG signal of CO adsorbed on platinum particles of 45 nm diameter was 4 orders of magnitude larger than that from CO on smooth platinum films.^75^ The local plasmon resonance peak of gold nanoparticles with a diameter of 40 nm is ∼545 nm, in line with the impinging visible pump beam.^81^ Conversely, the contribution from the IR electric field can be neglected, since the IR wavelength (generally 2.3–10 μm) is far away from the LSPR.^80^

However, while no surface-enhanced SFG on Ir nanoparticles was observed so far, LSPR induced by Ir metallic nanostructures has been extensively reported. More specifically, the performance of diamond photodetectors was significantly enhanced due to the LSPR induced by Ir nanoislands,^83^ with 210 nm UV light being far more effective than 420 nm visible light illumination. Conversely, no LSPR effect was observed under 532 nm laser excitation, as the intensity and width of the Raman peak of diamond (1331.42 cm^–1^) showed minimal change. Using 532 nm laser radiation, a strong surface-enhanced Raman scattering (SERS) activity, with an enhancement factor of 3.5 × 10^5^ at the 1512 cm^–1^ peak, was observed on 2.5 nm colloidal Ir nanoparticles (IrNPs) due to their LSPR, using R6G as the probe molecule.^84^ The Ir NPs displayed absorption bands at 250, 400, and 600 nm. Moreover, by varying the shape, size, and material of the nanostructures and, thus, by varying the surface plasmon energy, the LSPR wavelength can be tuned throughout the visible spectral range.^55,57^ Summarizing, from the above findings it is reasonable to assume that upon sputtering an Ir(111) surface and forming plenty of grains 3–8 nm in size (Figure 2c–e), LSPR can be excited by the visible laser beam, resulting in an intensity enhancement in both ppp and ssp vibrational spectra. At this point, we thus conclude that LSPR effects account for the observed phenomenon. To study this in more detail, doubly resonant SFG would be beneficial as it can tune both the IR and visible frequency.^2,85–90^

## 5 Conclusions

Polarization-dependent, ppp- and ssp-SFG spectra of CO adsorbed on smooth Ir(111) and rough sputtered Ir surfaces were acquired at room temperature. A significant enhancement in the CO-related SFG intensity was observed for rough Ir surfaces, a finding confirmed by exploiting two different SFG spectrometers and Ir(111) samples. This enhancement was also observed in ref 4 both for various CO coverages and substrate temperatures. From the perspective of fundamental SFG theory, a smaller CO tilt angle on the rough surface (i.e., a preferential upright orientation) led to intensity enhancement, but only to a small extent. Most likely, the major contribution to the intensity enhancement is via a localized surface plasmon in resonance with the visible pump beam radiation, due to changes in the electronic structure resulting from the sputter-induced local surface roughness. Future surface-enhanced Raman, IR or SFG spectra of CO adsorption on Ir nanoparticles may corroborate our findings. Moreover, to address a potential increase of molecular hyperpolarizability (β_ccc_^(2)^) on the rough surface, which would cause both higher ppp and ssp signals (eqs 1 and 2), further theoretical calculations are needed.

## Supplementary Material



## Data Availability

All data that support the findings of this study are included within the article (and any Supplementary Files).

## Notes

ppp-SFG spectra of CO on smooth and rough Ir(111) acquired at various CO pressures at TU Wien (PDF)
