# Supplementary material for: Local Geometry, Structure and Electronic Resonances Enhancing the SFG Signal from CO on Ir Surfaces
Source: J Phys Chem C Nanomater Interfaces. 2025 Jul 1;129(27):12551–60. doi: 10.1021/acs.jpcc.5c02545 (PMC12257592; doi:10.1021/acs.jpcc.5c02545)
Supplement: Supplementary file 1 [file jp5c02545_si_001.pdf]

Local Geometry, Structure and Electronic Resonances Enhancing the SFG Signal from CO on Ir Surfaces

Xia Li[a], Stefania Baronio[b], Susanne Gross[a], Thomas Haunold[a], Erik Vesselli[b,c], Günther Rupprechter[a]*

[a]Institute of Materials Chemistry, Technische Universität Wien, Getreidemarkt 9/BC, 1060 Vienna, Austria.

[b]Physics Department, University of Trieste, via Valerio 2, I-34127 Trieste, Italy.

[c]CNR - Istituto Officina dei Materiali (IOM), Area Science Park, SS 14 km 163.5, I-34149 Basovizza (Trieste), Italy.

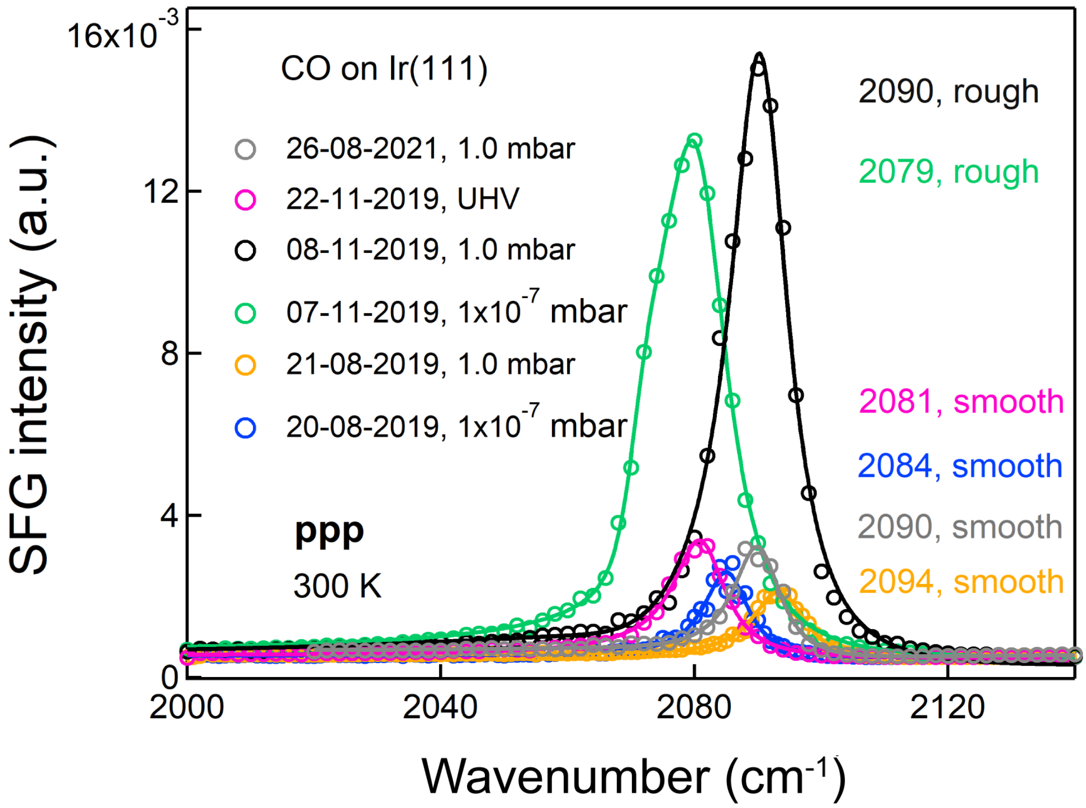

Figure S1 *ppp-SFG spectra of CO on smooth and rough Ir(111) acquired at various CO pressures at TU Wien.*
